# Depressive symptoms and their determinants in patients who are on antiretroviral therapy in the case of a low-income country, Ethiopia: a systematic review and meta-analysis

**DOI:** 10.1186/s13033-020-00430-2

**Published:** 2021-01-06

**Authors:** Mogesie Necho, Asmare Belete, Mekonnen Tsehay

**Affiliations:** Department of Psychiatry, College of Medicine and Health Sciences, Wollo University, Dessie, Ethiopia

**Keywords:** Meta-analysis, Depression, HIV in Ethiopia

## Abstract

**Background:**

The presence of depression in people living with HIV/AIDS could lead to non-adherence to antiretroviral medications. It also leads to further comorbid and opportunistic illness and then lowering the patient's quality of life. The objective of this study was therefore to determine the pooled prevalence of depression and its related factors in HIV patients.

**Methods:**

Relevant articles in PubMed, Scopus, and EMBASE were investigated. The Meta-XL version 5.3 was used to extract data and STATA-11 Meta-prop packages with the Random effect model was used to quantify depression and its related factors. Sensitivity and subgroup analysis were performed to explore sources of heterogeneity. The Cochran’s Q-statistic and the Higgs I^2^ test were also done. Besides, the Eggers test and symmetry in the funnel plot were used to detect the presence/absence of publication bias.

**Result:**

In this meta-analysis, we included 21 articles that assessed 10,090 participants. The average prevalence of depression among people with HIV/AIDS was 35.8% (95% CI 28.29, 43.25). The average estimated prevalence of depressive symptoms was 59.4% in the Oromia region and 29.25% in southern Ethiopia. Besides, the average prevalence of depression was 45.6% and 26.2% as measured with Beck’s depression inventory and Hospital anxiety and depression scale respectively. Moreover, the prevalence of depression was 47.7% in studies that used a sample size ≤ of 400 participants and 28.5% in studies that used a sample size of > 400 participants. The pooled adjusted odds ratio (AOR) of perceived HIV stigma was 3.75 (95% CI 2.34, 5.16) and that of poor social support was 6.22 (95% CI 2.96, 9.47). Moreover, the average odds ratio of poor medication adherence, opportunistic infection, and advanced stages of AIDS were 3.03 (95% CI 1.00, 5.05), 5.5 (95% CI 1.97, 10.03), and 5.43 (95% CI 1.60, 9.28) respectively.

**Conclusion:**

The pooled prevalence of depression among individuals living with HIV/AIDS was high. Factors such as perceived HIV stigma, poor social support, opportunistic infection, advanced AIDS stage, and poor medication adherence were related to it. Routine screening and management of depression and its related factors should be given due consideration.

## Background

Depression is a psychiatric disorder with clinical presentations of depressed mood, loss of pleasure, reduced motivation, and energy, guilty feelings or low self-esteem, troubled sleep or appetite, suicidal thoughts, and concentration difficulties. Currently, more than 350 million people are living with depression [1]. Globally, it is the fourth leading cause of disability and is the second leading cause of disability with its lifetime prevalence in the general population estimated to be approximately 3 to 17% [2]. Research showed that depression is three times as high as more common in HIV-positive individuals and its lifetime prevalence in this population was estimated to be between 22 and 45% [3].

A systematic review and meta-analysis study by Uthman 2014 [4] found that the prevalence of depression in HIV-positive people in low-, middle-, and high-income countries range from 12.8% to 78%. Another systematic review study done in sub-Saharan Africa on depression among HIV patients incorporated 30 studies and a total of 10,000 participants from 10 countries. The reported pooled estimated occurrence of depression in this study was 31.2% [5]. Similarly, a study in sub-Saharan Africa reported the prevalence of depression range from 9 to 32% [6]. Furthermore, a meta-analysis of depression on east African HIV patients by Ayano et al. [7] revealed that the pooled prevalence of depression in HIV/AIDs patients was 38%. Other individual studies from different countries reported that depression in HIV positive individuals was 28.1% in France [8], 40% in India [9], 37.6% in South Africa [10], 47% in Uganda [11], 56.7% in Nigeria [12], 28% in Kenya, Tanzania and Namibia together [13].

In Ethiopia the reported prevalence of depression in HIV positive individuals ranges from 13.1 to 47.2% in Addis Ababa [14–19], 7.3% to 60% in Amhara region [18, 20–23], 11.2% to 48.0.6% in southern nations and nationalities of Ethiopia [24–27], 45.8% to 76.7% in Oromia region [28–30] and 14.9% to 57.9% in Tigray region [31–33]. Many factors were responsible for such high prevalence of depression in HIV patients. These includes the presence of perceived HIV stigma [16, 19, 22, 23, 26, 27], poor social support [16, 19, 23, 24, 26, 27], poor medication adherence [16, 19, 30, 32],the presence of opportunistic infection [19, 26, 29] and being in the advanced stage of AIDS [16, 22, 32].

The impacts of depression in people living with the human immune deficiency virus include poor quality of life [34], poor social conditions, poor compliance with prescribed treatment, poor therapeutic outcomes, and risky behaviors [35–37]. Furthermore, it predisposes to additional medical and psychiatric problems [38], unemployment, and disability [39–41].

Evidence showed that Sub-Saharan Africa countries in general and Ethiopia, in particular, are among the most vulnerable populations to be affected by the high prevalence of HIV AIDS.

Despite this, the mental health aspect of the population living with HIV in general and depression, in particular, were not addressed well. Even, those studies done are single studies that might not be as strong to be generalizable. Therefore, this systematic review and meta-analysis study was aimed to have aggregate empirical evidence on (1) The prevalence of depression in people living with HIV AIDS in Ethiopia and (2) The associated factors for the co-occurrence of depression in people with HIV AIDS and (3) To articulate a recommendation for policymakers, future researchers and clinicians in line with its findings.

## Methods

This Preferred Reporting Items for Systematic Reviews and Meta-Analyses (PRISMA) guidelines [42] had been utilized as a reference in conducting this study. We practically performed the search strategy for this review in the following two steps. The first step was the exploration of different databases (PubMed, Scopus, and EMBASE) to retrieve scientific evidence regarding depressive symptoms and its related factors in HIV/AIDS patients. The search strategy in the PubMed database was performed using the following key terms and words Epidemiology OR prevalence OR magnitude OR incidence AND depressive symptoms OR depression OR depressive disorder OR depressive AND HIV OR human immunodeficiency virus OR AIDS OR PLWHA OR ART AND factor OR risk OR risk factor OR determinant AND Ethiopia. Besides, we searched EMBASE and Scopus databases following the specific guideline of each database. The next step was a manual search for the reference list of included studies. During the search process, we did not put restrictions on the year of publication of the articles. For the determinants of depression in individuals who live with HIV/ADIS, narrative description, as well as the magnitude of pooled adjusted odds ratio, were employed.

### Eligibility criteria

An article was eligible for inclusion in the analysis if it fulfills the following criteria’s: (1) The initial criteria was that the study must assess depression in adults HIV patients, (2) the study design should be either, cross-sectional, cohort, or case–control design, (3) the outcome investigated should be depression, (4) studies should assess the associated factors for depression and (5) the study must be conducted in Ethiopia. Previous reviews, studies on non-human subjects, editorials, and articles published in non-English language were excluded. Initially, MN and AB individually screened articles stored in an endnote reference manager based on title and abstract. In the next step, these authors fully read the content of the articles that passed in the initial step, and independently decided on the articles that had to be included for final meta-analysis. Any differences between these authors concerning inclusion/exclusion criteria were solved by consensus and discussion with a third author (MT).

### Methods for data extraction and quality assessment

The previously listed authors (MN and AB) extracted the relevant information independently from the included articles using a standardized data extraction template developed by all of the authors at the beginning. The parameters included in the data extraction template and summary of articles were the author's name, publication year, study setting, study population, sample size, study design, and the assessment instrument for depression in HIV patients. All eligible articles included in the final analysis were summarized in the form of a table. PRISMA guideline [42] has been used as a standard reference during the extraction of data from all of the included studies. We also used the modified Newcastle–Ottawa Scale (NOS) [43] during the quality assessment of studies included in the final analysis. The dimensions of the NOS scale includes representativeness of sample used and sample size, comparability between participants, statistical quality, and ascertainment of cases.

### Data synthesis and analysis

In this meta-analysis, the pooled prevalence of depressive symptoms and its associated factors with their 95% CIs were computed using a random-effects model [44]. The Meta-XL version 5.3 [45] and the STATA-11 Meta-prop package [46] were applied during the analysis.

Heterogeneity between the included studies was assessed with the Cochran *Q-*statistics and the Higgs *I*^2^ test [47]. The *I*^2^ value of zero defines the absence of heterogeneity and *I*^2^ values of 25, 50, and 75% signify little, moderate, and high heterogeneity respectively [47]. Since the study had substantial heterogeneity, we further conducted a sensitivity analysis to know whether the result was influenced by a single study. Furthermore, a subgroup analysis was done by the region, measurement instrument used, and sample size. An eyeball funnel plot test [48] and Eggers regression test were also employed to identify publication bias. During this study, any statistical analysis with a P-value < 0.05 was interpreted as statistically significant.

## Results

### Identification of studies

**Fig. 1:**
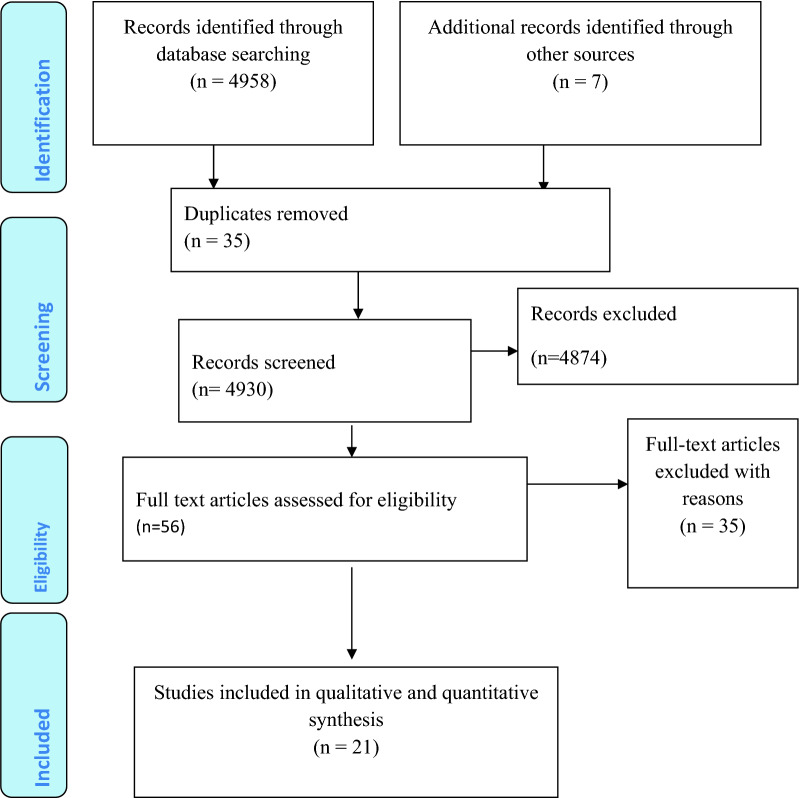
PRISMA flow chart for the review search process

Based on the predefined search strategies, a total of 4958 literature were identified. Additionally, 7 articles were identified by a manual search for the reference lists of other articles. This makes the overall search result to be 4965 articles. Of these 35 were duplicates and therefore removed. After further screening, only 56 articles were eligible for full-text revision. Finally, only twenty-one eligible articles that fulfilled our pre-specified inclusion criteria were incorporated in the analysis (Fig. 1).

### Characteristics of included studies

A total of 21 articles in Ethiopia [14–33, 49, 50] that examined depressive symptoms or its associated factors in 10,090 HIV positive individuals have been included in this systematic review and meta-analysis study. Regarding the regional distribution of the articles; six [14–19] were done in Addis Ababa, five [18, 20–23] were from the Amhara region, another four [24–27] from southern nations and nationalities of Ethiopia, one [30] from Harar, two [28, 29] from Oromia Region and the remaining three [31–33] from Tigray region.

Based on the study design, 19 of the included studies were cross-sectional, one cohort [20], and one case–control [33]. Among these 21 studies, 13 studies [14–18, 22–25, 27, 30, 32, 33] used a sample size greater than 400 participants, and the remaining eight studies [19–21, 26, 29, 31, 33, 49] used samples less than or equal to 400 subjects.

More than half of the included studies [17, 18, 21–23, 25–27, 30, 32, 33, 49] used PHQ-9 to screen depression in people living with HIV. HADS, CES-D, BDI, HAM-D, and Kessler were also used by two [16, 24], three [14, 20, 29], two [19, 50], one [31] and one [15] studies respectively. In addition, among all studies, 11 [16–18, 20, 21, 25–27, 29, 32, 33, 51] were published after 2015, and the remaining 10 studies [14, 15, 22–24, 28, 30, 31, 49, 50] were published before 2016 (Table 1).

**Table 1:** Characteristics of studies on depressive symptoms among HIV/AIDS patients which are incorporated in the meta-analysis according to author first name, year of publication, setting of study, design, sample size, assessment instrument, study population and magnitude of depression

Author, year	Study setting	Study design	Sample size	Tool (cutoff point)	Study population	Outcome variable	Depression (%)	Number of cases (n)
Abadiga et al. 2019	Gimbi-southern Ethiopia	CS	393	PHQ-9	Patients with HIV/AIDS	Depression	41.7	164
Duko et al. 2018	Hawassa, southern Ethiopia	CS	401	PHQ-9	Patients with HIV/AIDS	Depression	48.6	195
Eshetu et al. 2015	Debrebirhan, Amhara Ethiopia	CS	416	PHQ-9	Patients with HIV/AIDS	Depression	38.9	162
Bitew et al. 2016	Debark, North-west Ethiopia	CS	393	PHQ-9	Patients with HIV/AIDS	Depression	37.9	149
Wondie et al. 2019	Addis-Ababa, Ethiopia	CS	413	PHQ-9	Patients with HIV/AIDS	Depression	31.7	131
Gebremariam et al. 2017	Addis-Ababa, Ethiopia	CS	423	PHQ-9	Patients with HIV/AIDS	Depression	47.2	197
Tesfaw et al. 2016	Addis Ababa, Ethiopia	CS	417	HADS	Patients with HIV/AIDS	Depression	41.2	172
Mohammed et al. 2015	Harar, Eastern Ethiopia	CS	740	PHQ-9	Patients with HIV/AIDS	Depression	45.8	339
Dejenu 2015	Debremarkos, north west,Ethiopia	CS	412	PHQ-9	Patients with HIV/AIDS	Depression	11.7	48
Berhe and Bayray 2013	Tigray, Ethiopia	CS	269	HAM-D	Patients with HIV/AIDS	Depression	43.9	118
Solomon and Girma 2014	Dila, SNNP	CS	500	HADS	Patients with HIV/AIDS	Depression	11.2	56
Yeneabat et al. 2017	Fiche, oromia	CS	390	CES-D Tool	Patients with HIV/AIDS	Depression	76.7	299
Gesbreegziabher et al. 2019	Aksum, Ethiopia	CS	411	PHQ-9	Patients with HIV/AIDS	Depression	14.6	60
Abebe et al. 2019	Addis Ababa, Ethiopia	CS	507	BDI	Patients with HIV/AIDS	Depression	35.5	180
Mekuriaw et al. 2015	Addis Ababa, Ethiopia	CS	664	Kessler-6	Patients with HIV/AIDS	Depression	15	99
Yakob 2015	SNNP, Ethiopia	CS	485	PHQ-9	Patients with HIV/AIDS	Depression	15.5	75
Weldehaweria 2017	Tigray, Ethiopia	Case control	340	PHQ-9	Patients with HIV/AIDS	Depression	57.9	197
Alemu 2012	Addis Ababa, Ethiopia	CS	1815	CES-D	Patients with HIV/AIDS	Depression	13.1	238
Bezabih et al. 2016	Amhara, Ethiopia	Cohort	246	CES-D	Patients with HIV/AIDS	Depression	7.3	18
Endeshaw et al. 2014	Amhara,Ethiopia	CS	55	PHQ-9	Patients with HIV/AIDS	Depression	60	33
Amberbir et al. 2008	Oromia, Ethiopia	CS	400	BDI	Patients with HIV/AIDS	Depression	55.8	223

*AIDS* Acquired Immune Deficiency Syndrome, *BDI* Beck Depression Inventory, *CS* cross-sectional, *CES-D* Center for Epidemiological Studies Depression Tool, *HADS* Hospital Anxiety and Depression Scale, *HAM-D*:Hamilton Depression Rating Scale, *HIV* Human Immune Virus, *PHQ-9* Patient Health Questionnaire-9

### Quality of included studies

In general, the overall quality assessment score of 21 included studies based on the Newcastle Ottawa quality assessment scale ranges from 6 to 10. Amongst all studies, twenty were found to have good methodological quality and the remaining 1 was having moderate quality. However, none of the studies was found to have poor quality**.**

### The pooled prevalence of depression among HIV/AIDS patients in Ethiopia

**Fig. 2:**
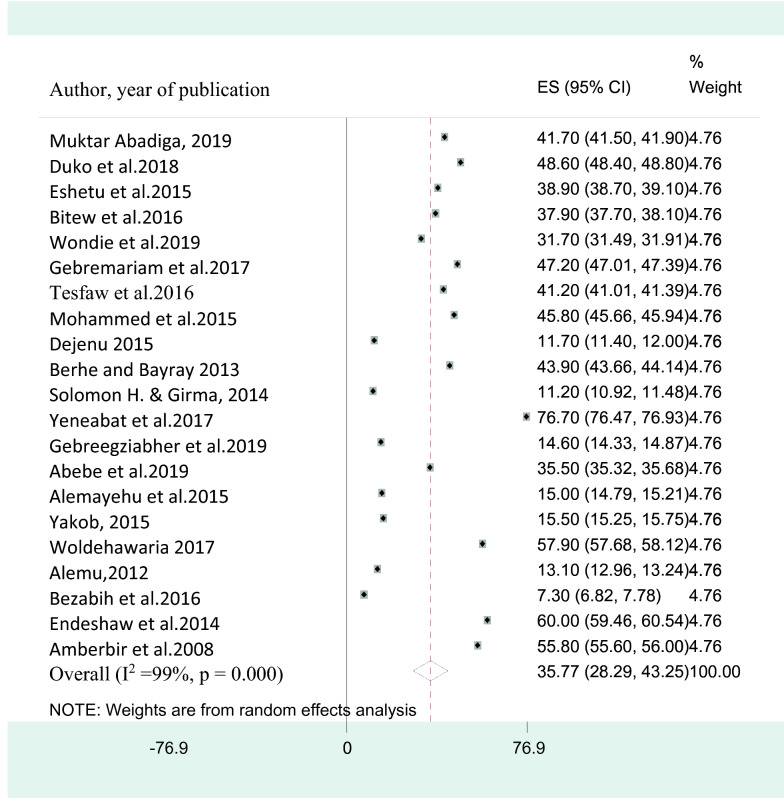
A forest plot for the prevalence of depressive symptoms in HIV/AIDS patients

Twenty-one studies [14–18, 20–26, 28–33, 49] in Ethiopia were pooled in the final meta-analysis to determine the average magnitude of depression among HIV positive individuals. The reported prevalence of depression in HIV AIDS patients among studies included in the analysis varies from as low as 7.3% in the Amhara region [20] to as high as 76.7%% in the Oromia region [29]. The pooled prevalence of depression among people with HIV AIDS in Ethiopia using the random effect model was 35.8% (95% CI 28.29, 43.25). This average estimate was influenced by a considerable heterogeneity (I^2^ = 99%, P-value < 0.001). Detailed information is presented in the forest plot of the pooled prevalence of depression among HIV patients in Ethiopia below (Fig. 2).

### Sub-group analysis of the prevalence of depression among HIV/AIDS patients in Ethiopia

Since the pooled prevalence of depression was influenced by substantial heterogeneity, we performed a subgroup analysis based on the regional location of the study, the measurement instrument used, the size of the sample studied, and the year of publication. Among included studies, six [14–19] were from Addis Ababa, five [18, 20–23] from Amhara region, four [24–27] from southern Ethiopia, three [28–30] from Oromia Region and the remaining three [31–33] from Tigray region. Based on this the pooled prevalence of depression was 30.6% (95% CI 18.82, 42.41) (I^2^ = 92.2%; P-value < 0.001) in Addis Ababa, 31.2% (95% CI 17.43, 44.89) (I^2^ = 96.2%; P-value < 0.001) in Amhara region, 29.3% (95% CI 11.45, 47.05) (I^2^ = 96%; P-value < 0.001) in southern region, 59.4% (95% CI 42.44, 76.42) (I^2^ = 99.5%; P-value < 0.001) in Oromia region and 38.8% (95% CI 14.66, 62.94) (I^2^ = 94.8%; P-value < 0.001) in Tigray region.

The pooled prevalence of depression among studies that utilized a sample of > 400 participants [14–18, 22–25, 27, 30, 32, 33] was 28.5% (95% CI 20.21, 36.72) (I^2^ = 96.8%; P-value < 0.001) whereas the prevalence in studies which assessed sample size ≤ 400 participants [19–21, 26, 29, 31, 33, 49] was 47.7% (95% CI 36.73, 58.57) (I^2^ = 99%; P-value < 0.001). The prevalence of depression was higher as measured with Beck depression inventory; 45.6% ((95% CI 25.76, 66.54) (I^2^ = 92.6%; P-value < 0.001). Furthermore, the pooled prevalence of depression among studies that utilized PHQ-9 screening tool [17, 18, 21–23, 25–27, 30, 32, 33, 49] was 37.6% (95% CI 30.05, 45.17) (I^2^ = 98.8%; p-value < 0.001) (Table 2).

**Table 2:** A subgroup analysis of the prevalence of depressive symptoms among HIV AIDS patients in Ethiopia with its 95% confidence interval

Subgroup	Number of studies	Estimates		Heterogeneity		
		Prevalence (%)	95% CI	I^2^	Q(DF)	P-value
Study setting						
North western Ethiopia (Amhara region)	5	31.16	17.43, 44.89	96.2%	185.27 (4)	P < 0.001
South western Ethiopia(Oromia region)	3	59.43	42.44, 76.42	99.5%	403.2 (2)	P < 0.001
Tigray region	3	38.8	14.66, 62.94	94.8%	212 (2)	P < 0.001
Central Ethiopia(Addis Ababa)	6	30.61	18.82, 42.41	92. 2%	95.43 (5)	P < 0.001
Southern Ethiopia	4	29.25	11.45, 47.05	96%	164.20 (3)	P < 0.001
Assessment tool used						
PHQ-9	12	37.62	30.05, 45.17	98.8%	521.45 (11)	P < 0.001
HADS	2	26.2	3.20, 55.60	94.6%	453.21 (1)	P < 0.001
CES-D	3	32.37	− 14.05, 78.79	96.4%	496.32 (2)	P < 0.001
BDI	2	45.6	25.76, 66.54	92.6%	243.40 (1)	P < 0.001
Others*	2	29.4	1.13, 55.77	98.6%	502.65 (1)	P < 0.001
Sample size studied						
≤ 400	8	47.7	36.73, 58.57	99%	232.21 (7)	P < 0.001
> 400	13	28.5	20.21, 36.72	96.8%	136.35 (12)	P < 0.001
Year of publication						
After 2015	11	40	31.17, 48.89	96.6%	221.22 (10)	P < 0.001
2015 and before	10	31.1	19.62, 42.56	98%	311.37 (9)	P < 0.001

Others* Includes HAM-D & Kessler-6, *DF* Degree of Freedom, *CI* confidence interval

### Sensitivity analysis

We performed one study leave out at a time sensitivity analysis and its result showed that the pooled estimated prevalence of depression obtained when each of the included studies was left out from the analysis at a time was within the 95% confidence limit of the pooled estimate of depression when all studies were pooled together. This suggested that the overall average prevalence was not influenced by a single particular study. The average prevalence of depression when each of the 21 studies was left out from the analysis ranges between 33.7% (95% CI 26.82, 40.63) and 37.2% (95% CI 29.60, 44.79) (Table 3).

**Table 3:** A sensitivity analysis of the prevalence depressive symptoms among HIV AIDS patients in Ethiopia when each indicated studies are removed at a time with its 95% confidence interval

No.	Study excluded	Prevalence of depression symptoms (%)	95% Confidence interval
1	Abadiga 2019	35.5	27.60, 43.35
2	Duko et al. 2018	35.1	27.34, 42.92
3	Eshetu et al. 2015	35.6	27.72, 43.51
4	Bitew et al. 2016	35.7	27.78, 43.55
5	Wondie et al. 2019	35.9	28.12, 43.83
6	Gebremariam et al. 2017	35.2	27.38, 43.02
7	Tesfaw et al. 2016	35.5	27.61, 43.39
8	Mohammed et al. 2015	35.3	27.31, 43.23
9	Dejenu 2015	36.9	29.41, 44.54
10	Berhe and Bayray 2013	35.4	27.57, 43.16
11	Solomon and Girma 2014	37	29.47, 44.53
12	Yeneabat et al. 2017	33.7	26.82, 40.63
13	Gebreegziabher et al. 2019	36.8	29.24, 44.42
14	Abebe et al. 2019	35.8	27.85, 43.72
15	Mekuriaw et al. 2015	36.8	29.26, 44.36
16	Yakob 2015	36.8	29.26, 44.36
17	Woldehawaria 2017	34.7	27.10, 42.23
18	Alemu 2012	36.9	29.83, 43.98
19	Bezabih et al. 2016	37.2	29.60, 44.79
20	Endeshaw et al. 2014	34.6	26.92, 42.20
21	Amberbir et al. 2008	34.8	27.17, 42.37

### Publication bias

**Fig. 3:**
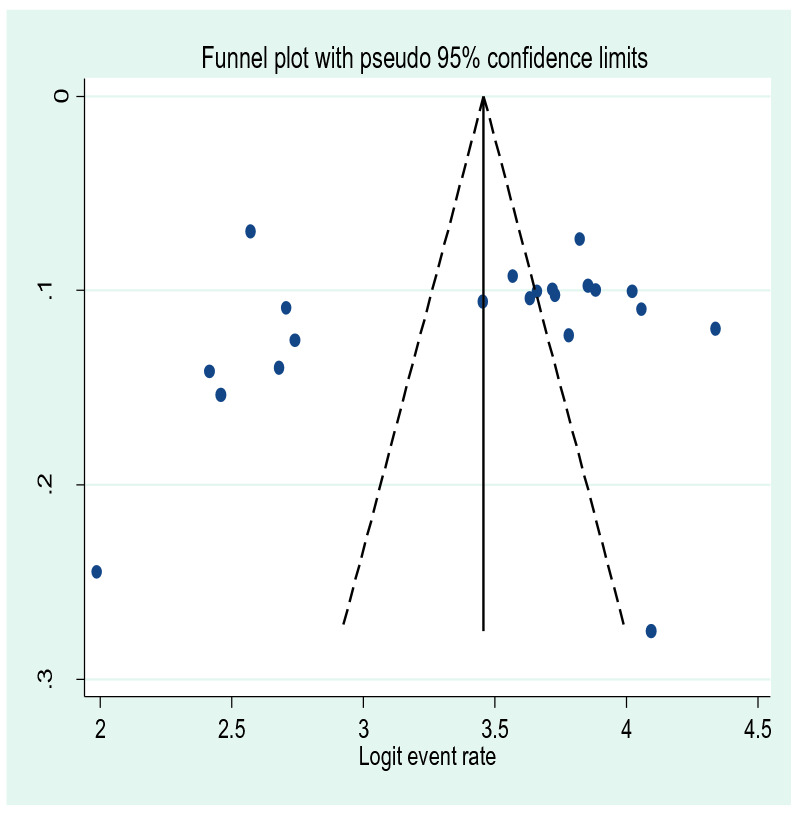
A funnel plot for the prevalence of depressive symptoms in HIV/AIDS patients

Egger's publication bias plot test was done to detect publication bias. However, there was no publication bias in this meta-analysis since the P-value of eggers publication bias plot test is insignificant (P-value = 0.86). This can also be strengthened with a visual inspection from a funnel plot for a Logit event rate of prevalence of depression in HIV AIDS patients against its standard error (Fig. 3).

### Associated factors for depression in HIV/AIDS patients in Ethiopia

Of all included studies, 11 [16, 19, 22–24, 26, 27, 29–32] reported data regarding the associated factors for depression in HIV positive individuals. Qualitatively, perceived HIV stigma, opportunistic infection, adverse drug reaction, poor social support, co-morbid chronic illness, being female, being on stage 4-AIDS, living alone, being unemployed, low income, negative life events, and non-disclosure of HIV status were among the factors found to have an association with the development of depression in HIV patients. However, the most commonly reported factors by the included studies were presence of perceived HIV stigma [16, 19, 22, 23, 26, 27], poor social support [16, 19, 23, 24, 26, 27], poor medication adherence [16, 19, 30, 32], opportunistic infection [19, 26, 29] and advanced stage of AIDS [16, 22, 32] (Table 4).

**Table 4:** Characteristics of associated factors for depression among HIV AIDS patients in Ethiopia by their Odds ratio, Confidence interval, association strength, author and year of publication

Associated factors	Odds ratio(AOR)	95% CI	Strength of association	Author, year of publication
Perceived stigma	6.98	3.07, 15.86	Strong and positive	Abadiga 2019
Opportunistic infection	9.38	4.21, 20.89	Strong and positive	Abadiga 2019
Adverse drug reaction	3.7	1.58, 8.81	Strong, positive	Abadiga 2019
Poor social support	9.9	3.57, 27.86	Strong and positive	Abadiga 2019
Co-morbid chronic illness	6.1	1.66, 22.68	Strong and positive	Abadiga 2019
Poor social support	2.5	1.70, 9.13	Strong and positive	Duko et al. 2018
Perceived HIV stigma	2.8	1.78, 4.48	Strong and positive	Duko et al. 2018
CD4 count < 200	3.9	1.02, 14.83	Strong and positive	Duko et al. 2018
Being female	2.07	1.08, 3.98	Strong and positive	Eshetu et al. 2015
30–39 years old	2.8	1.16, 6.54	Strong and positive	Eshetu et al. 2015
60–69 years old	19.6	4.02, 95.99	Strong and positive	Eshetu et al. 2015
Low income(< 200ETB)	3.9	1.14, 6.86	Strong and positive	Eshetu et al. 2015
Stage 3 AIDS	2.3	1.11, 4.85	Strong and positive	Eshetu et al. 2015
Stage 4 AIDS	8.8	1.93, 39.87	Strong and positive	Eshetu et al. 2015
Perceived HIV stigma	3.6	1.86, 6.95	Strong and positive	Eshetu et al. 2015
Living alone	2.5	1.19, 5.08	Strong and positive	Dejenu 2015
Perceived stigma	3.4	1.63, 7.19	Strong and positive	Dejenu 2015
Stop working	2.7	1.78, 6.33	Strong and positive	Dejenu 2015
Poor social support	10	1.91, 33.16	Strong and positive	Dejenu 2015
Being male	1.6	1.14, 2.34	Moderate and positive	Mohammed et al. 2015
Being widowed	3.1	1.70, 5.75	Strong and positive	Mohammed et al. 2015
Monthly income 500-1000ETB	1.9	1.16, 3.19	Strong and positive	Mohammed et al. 2015
Missing medication	5.3	2.58, 10.77	Strong and positive	Mohammed et al. 2015
Being teased, insulted or sworn at	2.3	1.22, 4.29	Strong and positive	Mohammed et al. 2015
Gossiped about	2.9	1.68, 5.31	Strong and positive	Mohammed et al. 2015
Perceived HIV stigma	3.6	2.23, 5.80	Strong and positive	Tesfaw et al. 2016
Poor social support	2	1.25, 3.27	Strong and positive	Tesfaw et al. 2016
HIV stage III	2.8	1.50, 5.21	Strong and positive	Tesfaw et al. 2016
Poor medication adherence	1.6	1.02, 2.55	Moderate and positive	Tesfaw et al. 2016
Urban residence	3.2	1.50, 6.65	Strong and positive	Berhe and Bayray 2013
Low income(< 200ETB)	4.4	1.35, 14.58	Strong and positive	Berhe and Bayray 2013
Unemployed	2.7	1.34, 5.57	Strong and positive	Berhe and Bayray 2013
Government employed	3.6	1.73, 7.30	Strong and positive	Berhe and Bayray 2013
Moderate stress	6.9	2.27, 20.81	Strong and positive	Solomon and Girma 2014
Poor social support	10.2	2.85, 36.29	Strong and positive	Solomon and Girma 2014
≥ 6 negative life events	3.9	1.77, 8.99	Strong and positive	Solomon and Girma 2014
Non- disclosure of HIV status	5.2	1.33, 20.62	Strong and positive	Solomon and Girma 2014
Low CD4 count (≤ 350)	3.5	1.62, 7.73	Strong and positive	Solomon and Girma 2014
Food insecurity	3.8	1.57, 9.32	Strong and positive	Yeneabat et al. 2017
Non-ownership of livestock	2.2	1.16, 4.10	Strong and positive	Yeneabat et al. 2017
Opportunistic infection	5.2	1.34, 20.16	Strong and positive	Yeneabat et al. 2017
Non-adherence to ART	3.3	1.44, 7.76	Strong and positive	Gebreegziabher et al. 2019
WHO-stage II and above	4.7	1.32, 16.51	Strong and positive	Gebreegziabher et al. 2019
Living alone	2.4	1.09, 5.43	Strong and positive	Gebreegziabher et al. 2019
Having side effects of ART drug	2.8	1.14, 6.78	Strong and positive	Gebreegziabher et al. 2019
20–24 years of age	2.2	1.33, 3.62	Strong and positive	Abebe et al. 2019
Opportunistic infection	1.9	1.15, 3.27	Strong and positive	Abebe et al. 2019
Poor medication adherence	1.8	1.03, 2.98	Strong and positive	Abebe et al. 2019
Poor social support	2.7	1.13, 2.64	Strong and positive	Abebe et al. 2019
Moderate social support	1.8	1.03, 2.98	Strong and positive	Abebe et al. 2019
Perceived HIV stigma	2.1	1.35, 4.14	Strong and positive	Abebe et al. 2019

*AIDS* Acquired Immune deficiency Syndrome, *ART* Anti-retroviral therapy, *ETB* Ethiopian birr

**Fig. 4:**
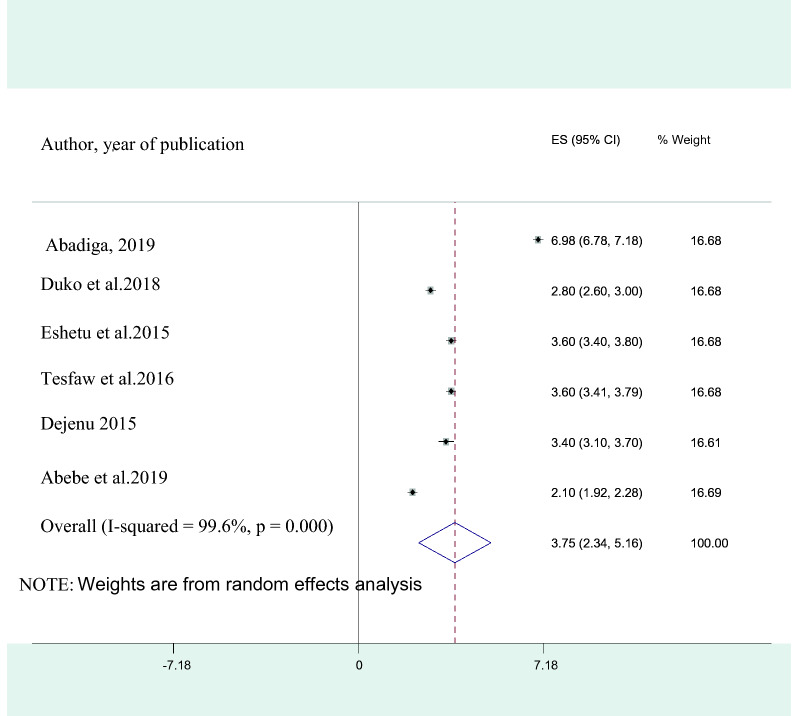
A forest plot for the perceived HIV stigma in HIV/AIDS patients

**Fig. 5:**
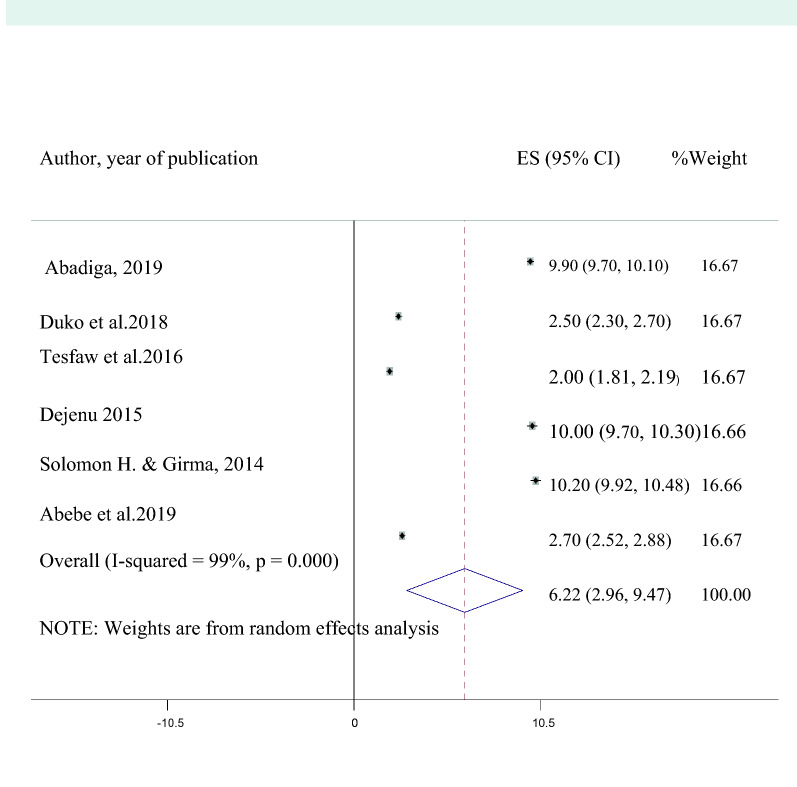
A forest plot for poor social support in HIV/AIDS patients

The pooled adjusted odds ratio (AOR) of perceived HIV stigma among the indicated studies was 3.75 (95% CI 2.34, 5.16). This implied that HIV positive individuals who have HIV related perceived stigma were 3.8 times more likely to develop depression than those who have not perceived HIV stigma (Fig. 4). Six studies also reported poor social support as an associated factor for depression in HIV patients and the pooled AOR was found to be 6.22 (95% CI 2.96, 9.47**)** which means that individuals with poor social support were 6.2 times at increased risk of developing than with good social support (Fig. 5). Moreover, the average odds ratio of poor medication adherence, presence of opportunistic infection, and advanced stages of AIDS were 3.03 (95% CI 1.00, 5.05), 5.5 (95% CI 1.97, 10.03), and 5.43 (95% CI 1.60, 9.28) respectively. Therefore HIV positive individuals with poor medication adherence, who have an opportunistic infection and who are in advanced stages of AIDS were 3, 5.5, and 5.4 times at a higher rate of developing depression as compared to those with good medication adherence, who have no opportunistic infection and in early stages of AIDS respectively.

## Discussion

The present meta-analysis study recommends the necessity of efforts to advance screening and management of depressive symptoms and its associated factors. Furthermore, researchers who are interested in assessing depression in HIV patients should use depression measures that exactly indicate the severity levels of depression which is best informative of the risk of the patient and figurative for further management. Moreover, it is imperative to have an improved understanding of depression services that could be integrated into the present ART therapy in Ethiopia.

To the knowledge of investigators, this meta-analysis study is the first of its kind to quantitatively assess both the prevalence as well as associated factors of depressive symptoms in HIV/AIDS patients in the context of Ethiopia. The main objective of the study was therefore to supplement epidemiological evidence concerning the magnitude of depressive symptoms and its related factors in HIV/AIDS patients. Consequently, the information obtained from this study on pooled magnitude and related factors for depressive symptoms would serve as an important baseline to a variety of stakeholders working in the area.

A total of twenty-one studies that assessed depressive symptoms and the related factors in Ethiopian HIV/AIDS patients were included in the analysis. The average estimated magnitude of depressive symptoms in this study was 35.8%. This was much higher than the pooled estimated prevalence of depressive symptoms in the general population in Ethiopia (9.1% to 11%) [52, 53]. This signifies the depression had a massive impact on the health of people with HIV/AIDS. This result of this meta-analysis is consistent with the result of a meta-analysis study by Ayano et.al. [7] in which the pooled magnitude of depressive symptoms in East African HIV/AIDS patients was 38%. However, a study on an average prevalence of depressive symptoms among Sub-Saharan HIV/AIDS patients as studied by Bernard et al. [6] obtained a relatively lower result (19%) when compared to the present study.

In this study, the pooled estimated prevalence of depressive symptoms varies based on the region of the country in which the studies were conducted, the sample size studied, the year of publication of the study, and the type of assessment tool used. In the current analysis, the average estimated prevalence of depressive symptoms in southwestern Ethiopia (Oromia region) (59.4%) was considerably greater than the pooled estimated prevalence in southern Ethiopia (29.3%). Region-specific cultural and socio-economic factors might be responsible for such variation. Moreover, factors related to the difference in ART facilities might also be responsible.

As anticipated, studies that utilized a larger sample size (> 400 samples) resulted in a lower pooled estimated depressive symptom (28.5%) than the pooled estimated depression prevalence of studies that used a smaller sample size (≤ 400);47.7%. This could be due to the minimal possibility of a standard error in larger sample size studies so that providing a more precise result that avoids overestimation of using a small sample.

There was also a substantial variety of pooled estimated depressive symptoms across the measurement instrument used. The average estimated prevalence of depression in HIV/AIDS patients as measured with PHQ-9, HADS, CES-D, BDI, and others (HAM-D&Kessler-6) was 37.6%, 26.2%, 32.4%, 45.6%, and 29.4% respectively. The smaller number of studies integrated into some of the assessment tool categories might minimize the estimate precision and responsible for the difference.

Concerning the associated factors for the occurrence of depressive symptoms in HIV/AIDS patients, a narrative description showed that perceived stigma, opportunistic infection, adverse drug reaction, poor social support, co-morbid chronic illness, being female, stage 4 AIDS, living alone, unemployed, low income, negative life events, and non-disclosure of HIV status were some of the associated factors which had a strong positive association with the development of depressive symptoms in HIV patients.

Moreover, quantitatively this meta-analysis investigated the pooled odds ratio of perceived HIV stigma, poor social support, poor medication adherence, advanced AIDS stage, and presence of opportunistic infection as they were the most frequently reported factors by the included studies. The pooled AOR of perceived HIV stigma among the included studies was 3.75. This implied that the presence of a perceived HIV stigma increases the probability of developing depressive symptoms by 3.8 times higher as compared with developing depressive symptoms in the absence of perceived stigma. This was strengthened by earlier meta-analysis studies [6, 7, 51]. The frequent psychological distress associated with perceived stigma might be responsible for this [16, 19, 22, 23, 26, 27].

Besides, the pooled AOR for studies that reported poor social support as a risk factor for depressive symptoms was 6.2. This means that having poor social support systems increasing the risk of developing depressive symptoms 6.2 times higher as comparatively seen with a good support system. This has also been supported by earlier studies and the reason might be HIV/AIDS patients with poor social support avoids disclosing about their problem due to social humiliation towards themselves, which further increases their separation and loneness [16, 19, 23, 24, 26, 27, 31] as well as decreasing help-seeking intention from professionals.

Moreover, the pooled AOR for advanced AIDS stage, medication non-adherence, and opportunistic infections in this analysis were 5.4, 3, and, 5.5 respectively. This suggests that HIV/AIDS patients with Advanced AIDS stage, Medication non-adherence, and opportunistic infections were 5.4, 3 and, 5.5 times at higher risk of developing depressive symptoms as compared to patients with early AIDS stage, good medication adherence, and no opportunistic infections respectively. Evidence in multiple kinds of literature strongly supports this conclusion.

### Difference between studies

This meta-analysis study on the prevalence of depressive symptoms and associated factors in HIV AIDS patients was potentially having a high degree of heterogeneity from the difference between the included studies. Therefore it was mandatory to further explore the source of such substantial heterogeneity. For this matter, a subgroup analysis had been done. The subgroup analysis result showed that the type of measurement instrument used to screen depression, the regional location at which the study was done, and the sample size utilized were responsible for the difference in the prevalence of depressive symptoms between included studies. Additionally, a single study leaves out at a time sensitivity analysis had also been performed but none of the studies were obtained to be influential on the overall estimate.

This meta-analysis study has some limitations. The primary limitation is that the few numbers of studies are included in a subgroup analysis might affect the precision of the estimate and result in either an overestimation or underestimation of pooled depression prevalence. Besides, the use of different study designs might result in the overestimation and underestimation of depression prevalence. Moreover, the presence of substantial heterogeneity in the pooled depression prevalence among HIV patients might greatly affect the validity of the data.

## Conclusion

This review and meta-analysis study found a high pooled prevalence of depression symptoms in Ethiopian people living with HIV/AIDS [35.8% (95% CI 28.29, 43.25)]. This average estimate for depressive symptoms had significant heterogeneity. The average estimated prevalence of depressive symptoms in the Oromia region (59.4%) was considerably higher than the pooled estimated prevalence in southern Ethiopia (29.3%), the Amhara region (31.2%), Central Ethiopia (30.6%), and the Tigray region (38.8%).

Besides, the average estimate was lower in studies that utilized a larger sample size (> 400 sample); (28.5%) than studies that used a smaller sample (≤ 400); 47.7%. Moreover, the average estimated prevalence of depression in HIV/AIDS patients as measured with PHQ-9, HADS, CES-D, BDI, and Others (HAM-D&Kessler-6) was 37.6%, 26.2%, 32.4%, 45.6%, and 29.4% respectively. The high pooled prevalence of perceived HIV stigma, poor social support, poor medication adherence, and the presence of opportunistic infections and advanced stages of AIDS increase the probability of developing depressive symptoms in HIV positive individuals in Ethiopia.

## Implications

The major implication of this meta-analysis study is the high occurrence of depression symptoms among HIV AIDS patients and the variety of contributing factors. This recommends the necessity of additional efforts to advance the screening and management of depressive symptoms and their associated factors. Furthermore, researchers who are interested in assessing depression in HIV patients should use depression measures that exactly indicate the severity levels of depression from mild depression to major depressive disorder which is best informative of the risk of the patient and figurative for further management. Moreover, it is imperative to have an improved understanding of depression services that could be integrated into the present ART therapy in Ethiopia (Additional file 1).

## Supplementary Information

**Additional file 1.** Quality assessment result of the studies included in this meta-analysis

## Data Availability

All available data concerning this study is included in the paper.

## Abbreviations

AIDS: Acquired Immune-deficiency Syndrome
AOR: Adjusted odds ratio
ART: Anti-retro viral therapy
BDI: Beck Depression Inventory
CES-D: Center for Epidemiological Studies Depression Scale
CI: Confidence Interval
HADS: Hospital Anxiety and Depression Scale
HAM-D: Hamilton Depression Rating Scale
HIV: Human Immune Virus
ICD-10: International Classification for Diseases-10
OR: Odds ratio
PHQ-9: Patient Health Questionnaire-9
PLWHA: People Living with HIV AIDS
PRISMA-P: Preferred Reporting Items for Systematic Reviews and Meta-analysis
