# Supplementary material for: Depressive symptoms and their determinants in patients who are on antiretroviral therapy in the case of a low-income country, Ethiopia: a systematic review and meta-analysis
Source: Int J Ment Health Syst. 2021 Jan 6;15:3. doi: 10.1186/s13033-020-00430-2 (PMC7789682; doi:10.1186/s13033-020-00430-2)
Supplement: Supplementary file 1 — Additional file 1. Quality assessment result of the studies included in this meta-analysis [file 13033_2020_430_MOESM1_ESM.docx]

**Additional file 1**: Quality assessment result of the studies included in this meta-analysis

| No | Study ID | Representation | sampling | Random selection | Non-response bias | Data collection | Case definition | Reliability and validity | Method of data collection | Prevalence period | Numerator and denominator | Summary |
| --- | --- | --- | --- | --- | --- | --- | --- | --- | --- | --- | --- | --- |
| 1 | Abadiga et al, 2019 | 1 | 0 | 1 | 1 | 1 | 1 | 1 | 1 | 1 | 0 | 8 |
| 2 | Duko et al.2018 | 1 | 1 | 1 | 1 | 1 | 1 | 1 | 1 | 1 | 1 | 10 |
| 3 | Eshetu et al.2015 | 1 | 1 | 1 | 0 | 1 | 1 | 1 | 1 | 1 | 0 | 8 |
| 4 | Bitew et al.2016 | 0 | 1 | 1 | 1 | 1 | 1 | 1 | 1 | 1 | 0 | 8 |
| 5 | Wondie et al.2019 | 1 | 1 | 1 | 1 | 1 | 1 | 1 | 1 | 1 | 1 | 10 |
| 6 | Gebremariam et al.2017 | 1 | 1 | 1 | 1 | 1 | 1 | 1 | 1 | 1 | 1 | 10 |
| 7 | Tesfaw et al.2016 | 1 | 1 | 1 | 1 | 1 | 1 | 1 | 1 | 1 | 1 | 10 |
| 8 | Mohammed et al.2015 | 1 | 1 | 1 | 1 | 1 | 1 | 1 | 1 | 1 | 1 | 10 |
| 9 | Dejenu 2015 | 1 | 1 | 1 | 0 | 1 | 1 | 1 | 1 | 1 | 1 | 9 |
| 10 | Berhe and Bayray 2013 | 0 | 1 | 1 | 0 | 1 | 1 | 1 | 1 | 1 | 1 | 8 |
| 11 | Solomon H. & Girma, 2014 | 0 | 1 | 1 | 1 | 1 | 1 | 1 | 1 | 1 | 1 | 9 |
| 12 | Yeneabat et al.2017 | 0 | 1 | 1 | 0 | 1 | 1 | 1 | 1 | 1 | 1 | 8 |
| 13 | Gesbreegziabher et al.2019 | 1 | 1 | 1 | 1 | 1 | 1 | 1 | 1 | 1 | 1 | 10 |
| 14 | Weldehaweria, 2017 | 0 | 1 | 1 | 0 | 1 | 1 | 1 | 1 | 1 | 1 | 8 |
| 15 | Mekuriaw et al.2015 | 1 | 1 | 1 | 1 | 1 | 1 | 1 | 1 | 1 | 1 | 10 |
| 16 | Yakob et al, 2015 | 1 | 1 | 1 | 1 | 1 | 1 | 1 | 1 | 1 | 1 | 10 |
| 17 | Abebe et al 2019 | 1 | 1 | 1 | 1 | 1 | 1 | 1 | 1 | 1 | 1 | 10 |
| 18 | Alemu,2012 | 1 | 1 | 1 | 0 | 1 | 1 | 1 | 1 | 1 | 1 | 9 |
| 19 | Bezabih et al.2016 | 0 | 1 | 1 | 0 | 1 | 1 | 1 | 1 | 1 | 1 | 8 |
| 20 | Endeshaw et al.2014 | 0 | 0 | 1 | 0 | 1 | 1 | 0 | 1 | 1 | 1 | 6 |
| 21 | Amberbir et al.2008 | 1 | 1 | 1 | 1 | 1 | 1 | 1 | 1 | 1 | 1 | 10 |

Yes=1, No=0

**The overall risk of bias scored based on the number of high risk of bias per study; a score of 8–10 indicating ‘low risk of bias and high quality study’, a score of 5–7 implies ‘moderate risk’ of bias and quality and 0–4: ‘high risk of bias and low quality study’.**

**Risk of bias assessment tool: Yes (low risk); No (high risk)**

1. **Representation**: Was the study population a close representation of the national population?

2. **Sampling**: Was the sampling frame a true or close representation of the target population?

3. **Random selection:** Was some form of random selection used to select the sample OR was a census undertaken?

4. **Non-response bias:** Was the likelihood of non-response bias minimal?

5. **Data collection**: Were data collected directly from the subjects?

6. **Case definition**: Was an acceptable case definition used in the study?

7. **Reliability and validity of study tool**: Was the study instrument that measured the parameter of interest show to have reliability and validity?

8**. Data collection**: Was the same mode of data collection used for all subjects?

9. **Prevalence period**: Was the length of the prevalence period for the parameter of interest appropriate?

10. **Numerators and denominators**: Were the numerator(s) and denominator(s) for the parameter of interest appropriate?
